# Infliximab Monotherapy as First-Line Treatment for Hepatic, Splenic, and Pulmonary Sarcoidosis in a Patient With High Cardiometabolic Risk

**DOI:** 10.7759/cureus.116827

**Published:** 2026-09-24

**Authors:** Matilde Coimbra, Carolina Saca, Maria Carolina Carvalho, Marisa Neves, José Delgado Alves

**Affiliations:** 1 Department of Internal Medicine IV, Hospital Professor Doutor Fernando Fonseca, Amadora, PRT; 2 Department of Internal Medicine, Hospital Professor Doutor Fernando Fonseca, Amadora, PRT; 3 Department of Internal Medicine IV, Systemic Immune-Mediated Diseases Unit (UDIMS), Hospital Professor Doutor Fernando Fonseca, Amadora, PRT

**Keywords:** anti-tnf therapies, cardiovascular disease risk, extra-pulmonary manifestations of sarcoidosis, infliximab, pulmonary sarcoidosis, steroid-sparing therapy

## Abstract

Glucocorticoids remain the first-line treatment for pulmonary and extrapulmonary sarcoidosis, with anti-tumor necrosis factor-alpha (TNF-α) agents such as infliximab typically reserved as steroid-sparing alternatives or for refractory disease. Data on anti-TNF-α monotherapy as first-line treatment, avoiding glucocorticoid exposure altogether, remain limited to isolated case reports. We report the case of a 46-year-old obese man with hypertension and dyslipidemia who presented with hepatic, splenic, and pulmonary sarcoidosis. Given his cardiometabolic risk profile, glucocorticoids were avoided entirely, and infliximab monotherapy was initiated as first-line treatment, resulting in marked clinical and radiological improvement within seven months. This case suggests that infliximab monotherapy may represent a safe and effective first-line alternative to glucocorticoids in selected patients with multiorgan sarcoidosis and significant cardiometabolic comorbidity.

## Introduction

Sarcoidosis is a multisystem granulomatous disease of unknown etiology, characterized histologically by non-caseating granulomas. Tumor necrosis factor-α (TNF-α), released by activated macrophages, is central to granuloma formation and maintenance, providing the rationale for TNF-α inhibition as a therapeutic target [1]. Infliximab is a monoclonal antibody that binds and neutralizes TNF-α; it is administered as an intravenous infusion.

Current guidelines recommend glucocorticoids as first-line therapy for both pulmonary and extrapulmonary sarcoidosis, with antimetabolites such as methotrexate used when a corticosteroid-sparing agent is required. Anti-TNF-α agents such as infliximab are generally reserved for refractory disease despite this combination or are used as glucocorticoid-sparing therapy in specific circumstances [2,3].

Prolonged corticosteroid exposure is itself a major source of morbidity in sarcoidosis, causing weight gain, dysglycemia, hypertension, and bone loss, adverse effects of particular concern in patients whose comorbidities already place them at high cardiometabolic risk [4,5].

Hepatic and splenic involvement, though less commonly discussed than pulmonary disease, occurs in a significant proportion of patients with systemic sarcoidosis and is not merely an incidental finding: splenic nodules can be associated with cytopenias, hypersplenism, and, rarely, splenic rupture, while hepatic granulomatous infiltration can progress to portal hypertension or, uncommonly, cirrhosis if left untreated. The presence of multiorgan involvement therefore carries prognostic weight and often signals a need for systemic, rather than purely symptomatic, therapy.

Whether anti-TNF-α therapy can be used as first-line monotherapy, bypassing both glucocorticoids and antimetabolites, in patients with significant cardiometabolic comorbidity remains largely unaddressed; the available evidence is limited to a small number of case reports [6].

We describe a patient with pulmonary, hepatic, and splenic sarcoidosis and significant cardiometabolic comorbidity in whom infliximab monotherapy was used as first-line treatment.

## Case presentation

A 46-year-old man presented with a six-month history of progressive fatigue. His medical history included obesity (body mass index 37.5 kg/m²), hypertension, and dyslipidemia. He was a non-smoker with no relevant occupational or environmental exposures. Physical examination was unremarkable. Laboratory studies revealed an elevated angiotensin-converting enzyme (ACE) level of 93.91 U/L (reference range, 8 to 53 U/L), with normal blood counts, thyroid function, serum calcium, and liver enzymes.

Contrast-enhanced CT revealed numerous small perilymphatic pulmonary nodules, enlarged mediastinal and hilar lymph nodes, multiple splenic nodules (up to 28 mm), and hepatic nodules (5 to 25 mm). A thorough oncological workup, including upper and lower gastrointestinal endoscopy, was negative for malignancy. After non-diagnostic sampling of the hilar lymph nodes (guided by endobronchial ultrasound) and a hepatic nodule (CT-guided), ultrasound-guided percutaneous biopsy of a splenic nodule showed non-caseating granulomas; special stains and cultures for mycobacteria and fungi were negative, supporting a diagnosis of sarcoidosis. Screening for latent tuberculosis, HIV, and viral hepatitis was negative. Cardiac and ophthalmologic evaluation showed no involvement.

Given the patient's metabolic comorbidities and the anticipated adverse effects of prolonged corticosteroid therapy, and after shared decision-making, glucocorticoids were withheld, and infliximab monotherapy was initiated (5 mg/kg at weeks 0, 2, and 6, and then every 8 weeks). After seven months, the patient reported marked improvement in fatigue, the ACE level normalized, and CT demonstrated partial resolution of the splenic and hepatic nodules and regression of the mediastinal lymphadenopathy (Figure 1). Treatment was well tolerated, without infusion reactions or infections.

**Figure 1:**
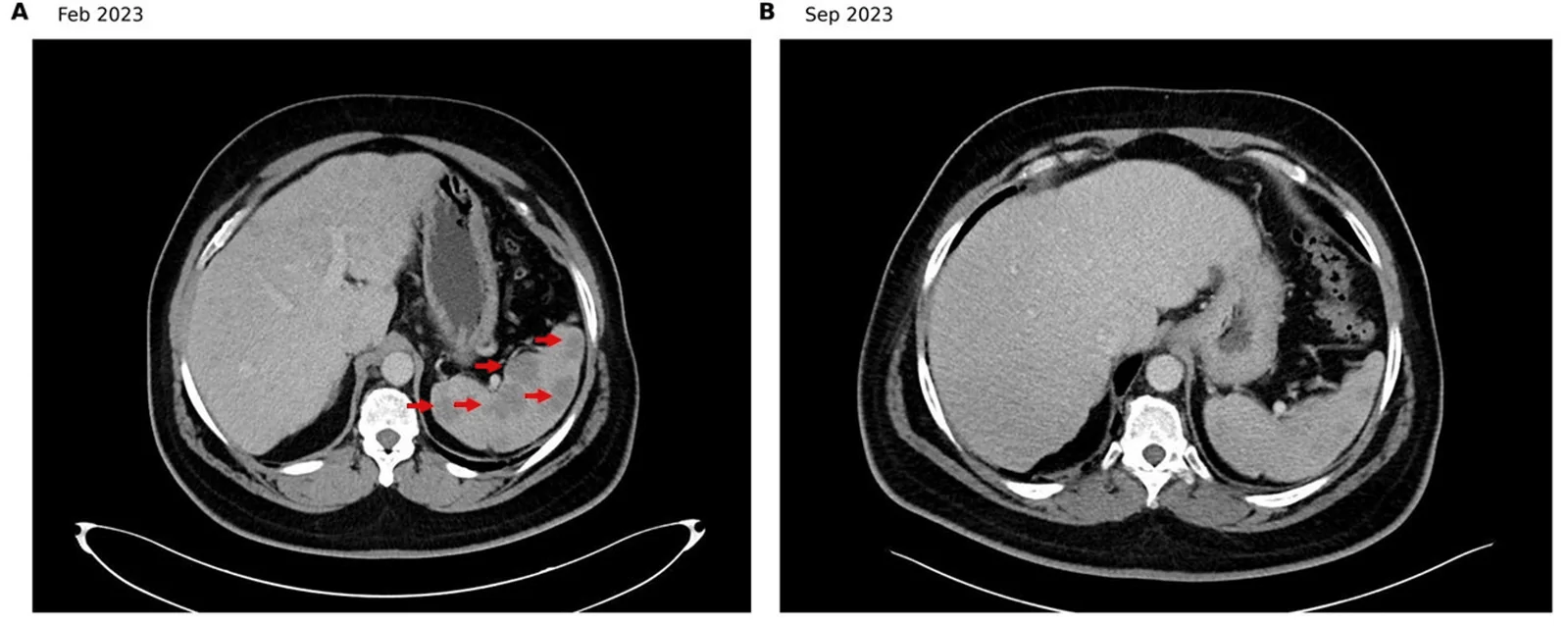
Evolution of splenic sarcoidosis after seven months of infliximab monotherapy (A) Contrast-enhanced axial CT of the upper abdomen at baseline (February 2023) showing multiple ill-defined hypoattenuating nodular lesions throughout the splenic parenchyma (red arrows). (B) Contrast-enhanced axial CT at the same anatomical level after seven months of infliximab monotherapy (September 2023), demonstrating partial resolution of the splenic nodules, with homogeneous parenchymal enhancement and few residual focal lesions. CT, computed tomography

## Discussion

This case illustrates two clinically relevant points: the diagnostic value of splenic biopsy when conventional sampling is non-diagnostic, and the potential role of infliximab monotherapy as first-line therapy in selected patients with sarcoidosis.

Mediastinal and hilar lymph nodes are the preferred biopsy targets in suspected sarcoidosis, and endobronchial ultrasound-guided sampling is the recommended first approach [7,8]. When this and other accessible sites are non-diagnostic, splenic biopsy is often avoided because of concerns regarding bleeding. In our patient, ultrasound-guided percutaneous splenic biopsy established the diagnosis without complications, avoiding a trial of empirical therapy without histological confirmation.

Current guidelines position glucocorticoids as first-line therapy, with anti-TNF-α therapy reserved for disease refractory to these treatments [2,3]. The recommended initial dose is typically up to 20 mg/day of prednisone equivalent; however, the duration of exposure is not clearly defined in the guidelines, as it depends on the evolution of the disease and the response to tapering, and treatment may extend well beyond the initially anticipated period. In patients with established cardiometabolic disease, this unpredictability warrants reconsideration: glucocorticoids predictably worsen hypertension, glycemic control, weight, and bone density, and the cumulative dose required to control multiorgan sarcoidosis is rarely trivial. Corticosteroid-related toxicity is a major contributor to morbidity and impaired quality of life in sarcoidosis [4,5,9], and fatigue, which was the dominant symptom in our patient, is itself frequently aggravated rather than relieved by corticosteroid therapy [10].

This reasoning has since been endorsed at the level of expert consensus: corticosteroids are no longer considered mandatory first-line therapy for all patients, and long-term corticosteroid use is now explicitly framed as an outcome to be avoided rather than an acceptable cost of disease control [11].

Infliximab monotherapy carries theoretical limitations, though none materialized in our patient over three years of follow-up. TNF-α inhibition has been linked to worsening cardiac function in patients with established moderate-to-severe heart failure (ATTACH trial, NYHA III-IV, LVEF ≤35%), a population distinct from ours; our patient had no baseline cardiac dysfunction and showed no cardiac impairment during treatment [12]. Monotherapy is also theoretically associated with higher rates of anti-drug antibody formation and secondary loss of response, a phenomenon well documented across infliximab-treated conditions and attributed to the immunogenicity of its chimeric structure; however, the disease remained controlled throughout, with no need for dose escalation or switching [13,14]. Unlike pulmonary disease, hepatic and splenic sarcoidosis lack standardized response biomarkers, though sustained radiological resolution over three years supported durable control. Finally, anti-TNF-α therapy requires baseline screening for latent tuberculosis and viral hepatitis and ongoing infectious surveillance, a predictable, manageable burden compared with the cumulative morbidity of prolonged glucocorticoid exposure.

To our knowledge, this is among the few reported cases of infliximab used as first-line monotherapy in sarcoidosis and illustrates that a corticosteroid-free strategy may be reasonable when comorbidities make glucocorticoid exposure particularly undesirable. Prospective studies are needed to determine whether upfront anti-TNF-α therapy improves outcomes in this subgroup and to define optimal treatment duration and discontinuation strategies.

## Conclusions

In this patient with multiorgan sarcoidosis and significant cardiometabolic comorbidity, first-line infliximab monotherapy was associated with clinical and radiological remission, obviating the need for systemic corticosteroid exposure. Splenic biopsy proved diagnostic after non-diagnostic sampling of more accessible sites. While a single observation cannot establish efficacy or safety, this case suggests that a corticosteroid-free strategy may be a reasonable option in selected patients for whom glucocorticoid toxicity is a particular concern. Prospective studies are needed to define which patients may benefit from upfront anti-TNF-α therapy.
